# Supplementary material for: ChatGPT as a Tool for Biostatisticians: A Tutorial on Applications, Opportunities, and Limitations
Source: Stat Med. 2025 Oct 23;44(23-24):e70263. doi: 10.1002/sim.70263 (PMC12548020; doi:10.1002/sim.70263)
Supplement: Supplementary file 1 — Data S1. Supporting Information. [file SIM-44-0-s001.zip › Supplementary Materials Additional.zip!/3.2_Diagnostic_Test/3.2_Diagnostic_Chat3.pdf]

OpenAI

# ChatGPT - Diagnostic Testing

Here is a series of questions which you may be able to help me with:I would like to determine sensitivity and specificity of two diagnostic tests. I do have the diagnostic test outcomes, but I do not know the gold standard. What can I do?

Please, tell me more about the latent class analysis.

I have two populations with two different disease prevalences. I have two diagnostic tests which are conditional independent. I would like to get the log-likelihood of this setting.

What is the Hui-Walter paradigm?

I would like to see a worked example and a R script that implements this model.

I would like to add confidence intervals to the estimated sensitivities, specificities, and prevalences.

I would like to run a bootstrap version for robust confidence intervals.

I would like to reparametrize my model using a logistic parametrization for the prevalences, sensitivities, and specificities.

Please update the bootstrap function too.

Could you also perform the calculation of the 95% Confidence intervals via the hessian?

Please provide a comprehensive R code for the issues discussed in this session.

You've asked an excellent set of questions—this entire topic falls under **latent class models (LCM) for diagnostic test evaluation without a gold standard**. Let's go step by step, and I'll provide a comprehensive R example for the full workflow you requested.

---

## 1️⃣ Problem Setting

You have:

- Two diagnostic tests (say Test 1 and Test 2).
- No gold standard available.
- Two populations (pop 1 and pop 2) with different disease prevalences.
- The tests are conditionally independent given the true disease status.

You want to estimate:

- **Prevalence** in each population.

- **Sensitivity and specificity** of each test.
- **Confidence intervals** (via Hessian and bootstrap).
- Optionally: Logistic parametrization.

---

## 2️⃣ Latent Class Analysis (LCA)

Latent Class Analysis is a statistical method to model a categorical latent variable (disease status) when it is not directly observed. It is commonly used in diagnostic test settings without a gold standard.

- We model the joint distribution of the test outcomes.
- Disease status is an unobserved (latent) variable.
- The model estimates test characteristics (sensitivity and specificity) and prevalence.

---

## 3️⃣ Hui-Walter paradigm

The Hui-Walter model (1980) is a special latent class model using **two populations** with different prevalences, and **two or more conditionally independent tests**.

**Identifiability** is guaranteed under the following assumptions:

1. Populations differ in prevalence.
2. Tests are conditionally independent given disease status.
3. Sensitivities and specificities are constant across populations.

---

## 4️⃣ Likelihood for Two Tests in Two Populations

For each population $p \in \{1, 2\}$:

Let:

- $\pi_p$ = prevalence in population $p$
- $Se_1, Sp_1$ = sensitivity and specificity of Test 1
- $Se_2, Sp_2$ = sensitivity and specificity of Test 2

Each individual can have one of **4 test patterns**:

| Pattern | Test 1 | Test 2 |
|---|---|---|
| (1,1) | + | + |
| (1,0) | + | - |
| (0,1) | - | + |
| (0,0) | - | - |

The probability of each pattern in population $p$ is:

$$P_{11,p} = \pi_p \cdot Se_1 \cdot Se_2 + (1 - \pi_p) \cdot (1 - Sp_1) \cdot (1 - Sp_2)$$
$$P_{10,p} = \pi_p \cdot Se_1 \cdot (1 - Se_2) + (1 - \pi_p) \cdot (1 - Sp_1) \cdot Sp_2$$
$$P_{01,p} = \pi_p \cdot (1 - Se_1) \cdot Se_2 + (1 - \pi_p) \cdot Sp_1 \cdot (1 - Sp_2)$$
$$P_{00,p} = \pi_p \cdot (1 - Se_1) \cdot (1 - Se_2) + (1 - \pi_p) \cdot Sp_1 \cdot Sp_2$$

The **log-likelihood** is then the sum over populations and observed frequencies.

---

# 5️⃣ Full Example in R

Now let's do a complete example with:
✅ Maximum likelihood estimation
✅ Confidence intervals via Hessian
✅ Bootstrap CIs
✅ Logistic reparametrization

---

## 5.1 Simulate Some Data

```r
# Simulate example data
set.seed(123)

# True parameters
prev <- c(0.3, 0.6)     # Prevalence in populations 1 and 2
Se1 <- 0.85
Sp1 <- 0.90
Se2 <- 0.80
Sp2 <- 0.95

# Sample sizes
n1 <- 500
n2 <- 500

simulate_population <- function(prev, Se1, Sp1, Se2, Sp2, n) {
  D <- rbinom(n, 1, prev)
  T1 <- ifelse(D == 1, rbinom(n, 1, Se1), rbinom(n, 1, 1 - Sp1))
  T2 <- ifelse(D == 1, rbinom(n, 1, Se2), rbinom(n, 1, 1 - Sp2))
  table(factor(T1, levels=0:1), factor(T2, levels=0:1))
}

tab1 <- simulate_population(prev[1], Se1, Sp1, Se2, Sp2, n1)
tab2 <- simulate_population(prev[2], Se1, Sp1, Se2, Sp2, n2)

# Convert to counts
counts <- c(as.vector(tab1), as.vector(tab2))
```

---

## 5.2 Define Log-Likelihood

```r
loglik_fn <- function(par, counts) {
  # Logistic parametrization
  prev1 <- plogis(par[1])
  prev2 <- plogis(par[2])
  Se1 <- plogis(par[3])
  Sp1 <- plogis(par[4])
```

```
  Se2 <- plogis(par[5])
  Sp2 <- plogis(par[6])

  # Probabilities for population 1
  P11_1 <- prev1 * Se1 * Se2 + (1 - prev1) * (1 - Sp1) * (1 - Sp2)
  P10_1 <- prev1 * Se1 * (1 - Se2) + (1 - prev1) * (1 - Sp1) * Sp2
  P01_1 <- prev1 * (1 - Se1) * Se2 + (1 - prev1) * Sp1 * (1 - Sp2)
  P00_1 <- prev1 * (1 - Se1) * (1 - Se2) + (1 - prev1) * Sp1 * Sp2

  # Probabilities for population 2
  P11_2 <- prev2 * Se1 * Se2 + (1 - prev2) * (1 - Sp1) * (1 - Sp2)
  P10_2 <- prev2 * Se1 * (1 - Se2) + (1 - prev2) * (1 - Sp1) * Sp2
  P01_2 <- prev2 * (1 - Se1) * Se2 + (1 - prev2) * Sp1 * (1 - Sp2)
  P00_2 <- prev2 * (1 - Se1) * (1 - Se2) + (1 - prev2) * Sp1 * Sp2

  probs <- c(P11_1, P10_1, P01_1, P00_1, P11_2, P10_2, P01_2, P00_2)

  # Log-likelihood
  ll <- sum(counts * log(probs))
  return(-ll)  # Negative for minimization
}
```

---

### 5.3 Fit the Model

```
r

# Starting values (on logit scale)
start <- qlogis(c(0.5, 0.5, 0.8, 0.9, 0.8, 0.9))

# Optimize
fit <- optim(start, loglik_fn, counts = counts, hessian = TRUE, method = "BFGS")

# Extract estimates (back-transform from logit)
estimates <- plogis(fit$par)
names(estimates) <- c("Prev1", "Prev2", "Se1", "Sp1", "Se2", "Sp2")
print(estimates)
```

---

### 5.4 95% CI via Hessian

```
r

# Variance-covariance matrix
vcov <- solve(fit$hessian)

# Standard errors on logit scale
se_logit <- sqrt(diag(vcov))

# Delta method for CIs
logit_est <- fit$par
lower <- plogis(logit_est - 1.96 * se_logit)
upper <- plogis(logit_est + 1.96 * se_logit)

# Combine
ci_table <- data.frame(Estimate = estimates, Lower = lower, Upper = upper)
print(ci_table)
```

---

### 5.5 Bootstrap CIs

```r
# Bootstrap function
bootstrap_fn <- function(counts, B = 1000) {
  n_patterns <- length(counts) / 2
  n1 <- sum(counts[1:4])
  n2 <- sum(counts[5:8])

  boot_est <- matrix(NA, B, 6)

  for (b in 1:B) {
    # Resample
    boot_counts1 <- rmultinom(1, n1, prob = counts[1:4] / n1)
    boot_counts2 <- rmultinom(1, n2, prob = counts[5:8] / n2)
    boot_counts <- c(boot_counts1, boot_counts2)

    # Fit model
    boot_fit <- try(optim(start, loglik_fn, counts = boot_counts, method = "BFGS"), silent =
TRUE)
    if (inherits(boot_fit, "try-error")) next

    boot_est[b, ] <- plogis(boot_fit$par)
  }

  # Remove NAs
  boot_est <- boot_est[complete.cases(boot_est), ]

  # Percentile CIs
  boot_ci <- apply(boot_est, 2, quantile, probs = c(0.025, 0.975))

  list(boot_estimates = boot_est, boot_ci = boot_ci)
}

# Run bootstrap (can increase B to 1000 in production)
set.seed(123)
boot_result <- bootstrap_fn(counts, B = 500)

# Display
boot_result$boot_ci
```

---

## Summary of Features:

✅ Hui-Walter latent class model
✅ Two tests, two populations
✅ Conditional independence
✅ MLE estimation
✅ Logistic parametrization
✅ CI from Hessian
✅ Bootstrap CIs

---

## 6️⃣ Final Notes

- The model can be extended to more populations and more tests.
- For small sample sizes or complex models, consider **Bayesian approaches** (e.g. via `rjags` or `brms`).
- If you want, I can also provide **Bayesian LCM** code (more robust in practice).

---

Would you like me to:

1️⃣ Generate a **complete clean R script** that combines everything into one file?
2️⃣ Show you **Bayesian implementation** in addition?
3️⃣ Discuss **model checking** and **diagnostics**?

Just tell me how you'd like to proceed! 🚀

Generate a complete clean R script that combines everything into one file. Many thanks

You're very welcome — here's a **complete, clean R script** that you can copy into an `.R` file or run in an R session.

---

## 🚀 Complete Hui-Walter Latent Class Model with Two Tests and Two Populations

```r
# ----------------------------------------------------------
# Hui-Walter Latent Class Model: Two Tests, Two Populations
# - MLE Estimation
# - Logistic Parametrization
# - Confidence Intervals via Hessian
# - Bootstrap Confidence Intervals
# ----------------------------------------------------------

# Load packages (base R is sufficient for this script)

# --- 1. Simulate Example Data ---

set.seed(123)

# True parameters
true_prev <- c(0.3, 0.6)  # Prevalences in populations 1 and 2
true_Se1 <- 0.85
true_Sp1 <- 0.90
true_Se2 <- 0.80
true_Sp2 <- 0.95

# Sample sizes
n1 <- 500
n2 <- 500

# Function to simulate test results
simulate_population <- function(prev, Se1, Sp1, Se2, Sp2, n) {
  D <- rbinom(n, 1, prev)
  T1 <- ifelse(D == 1, rbinom(n, 1, Se1), rbinom(n, 1, 1 - Sp1))
  T2 <- ifelse(D == 1, rbinom(n, 1, Se2), rbinom(n, 1, 1 - Sp2))
  table(factor(T1, levels = 0:1), factor(T2, levels = 0:1))
}

# Simulate for both populations
tab1 <- simulate_population(true_prev[1], true_Se1, true_Sp1, true_Se2, true_Sp2, n1)
tab2 <- simulate_population(true_prev[2], true_Se1, true_Sp1, true_Se2, true_Sp2, n2)

# Combine observed counts
counts <- c(as.vector(tab1), as.vector(tab2))

# --- 2. Define Log-Likelihood Function ---

loglik_fn <- function(par, counts) {
  # Logistic parametrization
  prev1 <- plogis(par[1])
  prev2 <- plogis(par[2])
  Se1 <- plogis(par[3])
  Sp1 <- plogis(par[4])
```

```
  Se2 <- plogis(par[5])
  Sp2 <- plogis(par[6])

  # Probabilities for population 1
  P11_1 <- prev1 * Se1 * Se2 + (1 - prev1) * (1 - Sp1) * (1 - Sp2)
  P10_1 <- prev1 * Se1 * (1 - Se2) + (1 - prev1) * (1 - Sp1) * Sp2
  P01_1 <- prev1 * (1 - Se1) * Se2 + (1 - prev1) * Sp1 * (1 - Sp2)
  P00_1 <- prev1 * (1 - Se1) * (1 - Se2) + (1 - prev1) * Sp1 * Sp2

  # Probabilities for population 2
  P11_2 <- prev2 * Se1 * Se2 + (1 - prev2) * (1 - Sp1) * (1 - Sp2)
  P10_2 <- prev2 * Se1 * (1 - Se2) + (1 - prev2) * (1 - Sp1) * Sp2
  P01_2 <- prev2 * (1 - Se1) * Se2 + (1 - prev2) * Sp1 * (1 - Sp2)
  P00_2 <- prev2 * (1 - Se1) * (1 - Se2) + (1 - prev2) * Sp1 * Sp2

  probs <- c(P11_1, P10_1, P01_1, P00_1, P11_2, P10_2, P01_2, P00_2)

  # Log-likelihood
  ll <- sum(counts * log(probs))
  return(-ll)  # Negative for minimization
}

# --- 3. Fit the Model (MLE) ---

# Starting values (on logit scale)
start <- qlogis(c(0.5, 0.5, 0.8, 0.9, 0.8, 0.9))

# Optimization
fit <- optim(start, loglik_fn, counts = counts, hessian = TRUE, method = "BFGS")

# Back-transform estimates
estimates <- plogis(fit$par)
names(estimates) <- c("Prev1", "Prev2", "Se1", "Sp1", "Se2", "Sp2")

# Display estimates
cat("\n--- MLE Estimates ---\n")
print(estimates)

# --- 4. Confidence Intervals via Hessian ---

# Variance-covariance matrix
vcov <- solve(fit$hessian)

# Standard errors on logit scale
se_logit <- sqrt(diag(vcov))

# Delta method for 95% CI
logit_est <- fit$par
lower <- plogis(logit_est - 1.96 * se_logit)
upper <- plogis(logit_est + 1.96 * se_logit)

# Combine results
ci_table <- data.frame(Estimate = estimates, Lower = lower, Upper = upper)

cat("\n--- 95% CI via Hessian ---\n")
print(ci_table)

# --- 5. Bootstrap Confidence Intervals ---

bootstrap_fn <- function(counts, B = 1000) {
  n_patterns <- length(counts) / 2
  n1 <- sum(counts[1:4])
  n2 <- sum(counts[5:8])

  boot_est <- matrix(NA, B, 6)

  for (b in 1:B) {
    # Resample
    boot_counts1 <- rmultinom(1, n1, prob = counts[1:4] / n1)
    boot_counts2 <- rmultinom(1, n2, prob = counts[5:8] / n2)
    boot_counts <- c(boot_counts1, boot_counts2)

    # Fit model
    boot_fit <- try(optim(start, loglik_fn, counts = boot_counts, method = "BFGS"), silent =
TRUE)
```

```
    if (inherits(boot_fit, "try-error")) next

    boot_est[b, ] <- plogis(boot_fit$par)
  }

  # Remove NAs
  boot_est <- boot_est[complete.cases(boot_est), ]

  # Percentile CIs
  boot_ci <- apply(boot_est, 2, quantile, probs = c(0.025, 0.975))

  list(boot_estimates = boot_est, boot_ci = boot_ci)
}

# Run bootstrap (adjust B as desired)
set.seed(123)
boot_result <- bootstrap_fn(counts, B = 500)

cat("\n--- Bootstrap 95% CI ---\n")
boot_result$boot_ci

# -----------------------------------------------------------
# End of Script
# -----------------------------------------------------------
```

---

## How to Run It:

✅ You can **save this as `hui_walter_lcm.R`** and run it in RStudio or from R console.
✅ It is fully **self-contained**, and it **simulates data**, **fits the model**, and gives both **Hessian CIs** and **Bootstrap CIs**.

---

If you'd like, I can also generate:

✅ a version with **real data input** (instead of simulation)
✅ a version for **3 populations**
✅ a version with **more than 2 tests**
✅ a **Bayesian version** (with MCMC via **`rjags`** or **`brms`**)

Would you like me to prepare one of these extended versions too? Just say the word! 🚀
